# Supplementary material for: HIV-1 Tat interactions with cellular 7SK and viral TAR RNAs identifies dual structural mimicry
Source: Nat Commun. 2018 Oct 15;9:4266. doi: 10.1038/s41467-018-06591-6 (PMC6189040; doi:10.1038/s41467-018-06591-6)
Supplement: Supplementary file 1 — Supplementary Information [file 41467_2018_6591_MOESM1_ESM.pdf]

Supplementary Table 1. DNA Template Sequences used for RNA synthesis

Name		Sequence
7SK-SL1^{apical}<br>(GAGA)	F	TAATACGACTCACTATAGGGATCTGTCACCCCATTGATCGCCGAGAGGCTGATC<br>TGGCTGGCTAGGCGGGTCCC
	R	GGGACCCGCCTAGCCAGCCAGATCAGCCTCTCGGCGATCAATGGGGTGACAGA<br>TCCCTATAGTGAGTCGTATTA
7SK-SL1^{apical}<br>(Native)	F	TAATACGACTCACTATAGGGATCTGTCACCCCATTGATCGCCAGGGTTGATTCG<br>GCTGATCTGGCTGGCTAGGCGGGTCCC
	R	GGGACCCGCCTAGCCAGCCAGATCAGCCGAATCAACCCTGGCGATCAATGGGG<br>TGACAGATCCCTATAGTGAGTCGTATTA
7SK-SL1^{apical}-<br>ASM$_1^{U76A}$	F	TAATACGACTCACTATAGGGATCTGTCACCCCATTGATCGCCGAGAGGCTGATC<br>TGGCTGGCAAGGCGGGTCCC
	R	GGGACCCGCCTTGCCAGCCAGATCAGCCTCTCGGCGATCAATGGGGTGACAGA<br>TCCCTATAGTGAGTCGTATTA
7SK-SL1^{apical}-<br>ASM$_2^{U72A}$	F	TAATACGACTCACTATAGGGATCTGTCACCCCATTGATCGCCGAGAGGCTGATC<br>TGGCAGGCTAGGCGGGTCCC
	R	GGGACCCGCCTAGCCTGCCAGATCAGCCTCTCGGCGATCAATGGGGTGACAGA<br>TCCCTATAGTGAGTCGTATTA
7SK-SL1^{apical}-<br>ASM$_3^{U40A}$	F	TAATACGACTCACTATAGGGATCTGTCACCCCAATGATCGCCGAGAGGCTGATC<br>TGGCTGGCTAGGCGGGTCCC
	R	GGGACCCGCCTAGCCAGCCAGATCAGCCTCTCGGCGATCATTGGGGTGACAGA<br>TCCCTATAGTGAGTCGTATTA
7SK-SL1^{apical}-<br>ASM$_4^{\Delta U63}$	F	TAATACGACTCACTATAGGGATCTGTCACCCCATTGATCGCCGAGAGGCGATCT<br>GGCTGGCTAGGCGGGTCCC
	R	GGGACCCGCCTAGCCAGCCAGATCGCCTCTCGGCGATCAATGGGGTGACAGAT<br>CCCTATAGTGAGTCGTATTA
7SK-SL1^{apical}-<br>$A_{39}G$, $U_{68}C$	F	TAATACGACTCACTATAGGGATCTGTCACCCCGTTGATCGCCGAGAGGCTGATC<br>CGGCTGGCTAGGCGGGTCCC
	R	GGGACCCGCCTAGCCAGCCGGATCAGCCTCTCGGCGATCAACGGGGTGACAGA<br>TCCCTATAGTGAGTCGTATTA
7SK-SL1^{top}	F	TAATACGACTCACTATAGGGCCCCATTGATCGCCCAGTGGGCTGATCTGGCTGG<br>CCC
	R	GGGCCAGCCAGATCAGCCCACTGGGCGATCAATGGGGCCCTATAGTGAGTCGT<br>ATTA
7SK-SL1^{bottom}	F	TAATACGACTCACTATAGGGATCTGTCACCCCAGGAGACTGGCTGGCTAGGCG<br>GGTCCC
	R	GGGACCCGCCTAGCCAGCCAGTCTCCTGGGGTGACAGATCCCTATAGTGAGTCG<br>TATTA
7SK-SL1^{top}-<br>$A_{39}G$, $U_{68}C$	F	TAATACGACTCACTATAGGGCCCCGTTGATCGCCCAGTGGGCTGATCCGGCTGG<br>CCC
	R	GGGCCAGCCGGATCAGCCCACTGGGCGATCAACGGGGCCCTATAGTGAGTCGT<br>ATTA
TAR	F	TAATACGACTCACTATAGGGCAGATTGAGCCTGGGAGCTCTCTGCCC
	R	GGGCAGAGAGCTCCCAGGCTCAATCTGCCCTATAGTGAGTCGTATTA

Supplementary Table 2. ITC derived binding parameters

Complex	N-value	K_D
TatRBD:7SK-SL1^{apical}(GAGA)	0.87 ± 0.02	32.2 ± 3.5 nM$^{§¢¥}$
TatRBD:7SK-SL1^{apical}(Native)	0.87 ± 0.02	51.8 ± 0.7 nM$^{§\infty€}$
Tat:CycT1:AFF4:7SK-SL1^{apical}(Native)	0.94 ± 0.10	55.3 ± 12.5 nM$^{*\infty}$
Tat:CycT1:AFF4:7SK-SL1^{apical}(GAGA)	1.04 ± 0.14	44.7 ± 15.4 nM*
TatRBD:7SK-SL1^{apical}-ASM_1^{U76A}	1.01 ± 0.21	308.7 ± 30.2 nM
TatRBD:7SK-SL1^{apical}-ASM_2^{U72A}	1.09 ± 0.03	86.8 ± 34.9 nM
TatRBD:7SK-SL1^{apical}-$ASM_4^{\Delta U63}$	1.01 ± 0.07	428.3 ± 221.4 nM
HEXIMRBD:7SK-SL1^{apical}(GAGA)	0.89 ± 0.02	66.6 ± 9.0 nM$^{£¢¥}$
HEXIMRBD:7SK-SL1^{apical}(Native)	0.89 ± 0.04	80.0 ± 1.8 nM$^{£€}$
TatRBD:TAR	0.92 ± 0.02	22.5 ± 15.2 nM^{+}
Tat:CycT1:AFF4:TAR	0.92 ± 0.04	77.7 ± 62.7 nM^{+}
TatRBD:7SK-SL1^{apical}-$A_{39}G$, $U_{68}C$	0.94 ± 0.02	140.5 ± 30.9 nM

*p=0.408, student t-test
$^{+}$p=0.351, student t-test
$^{\infty}$p=0.575, student t-test
$^{£}$p=0.918, student t-test
$^{¢}$p=0.002, student t-test
$^{¥}$p=0.037, student t-test
§p=0.027, student t-test

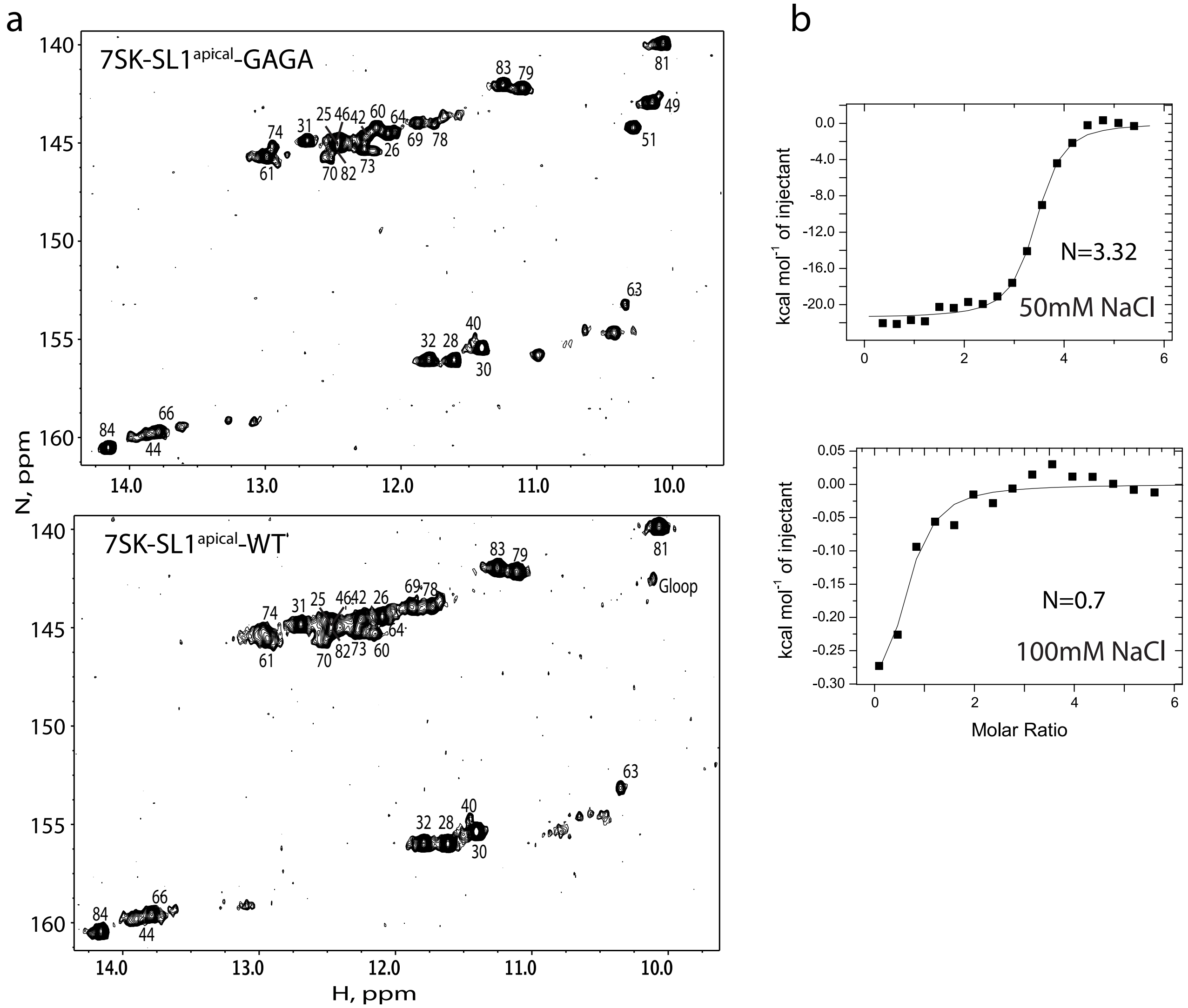

Supplementary Figure 1. Construct design and solution conditions for 7SK-SL1^{apical}:Tat RBD complex

(a) Overlay of two-dimensional ^{1}H–^{15}N HSQC spectra for the native (top) and GAGA loop (bottom) 7SK-SL1^{apical} constructs demonstrating that the residues in the stem are unaffected upon changing the loop. The data also shows that the pseudo-ASM$_3$ and ASM$_4$ that are closest to the loop remain unperturbed. **(b)** Representative ITC data for Tat RBD binding to 7SK-SL1^{apical} under different salt conditions, 50 mM NaCl (top) and 100 mM NaCl (bottom). A continuous line represents the fit for one set of sites binding model. Binding of Tat RBD under 50mM NaCl led to binding of three Tat RBD molecules to 7SK-SL1^{apical} whereas conditions greater than 70mM NaCl led to saturable, 1:1 binding.

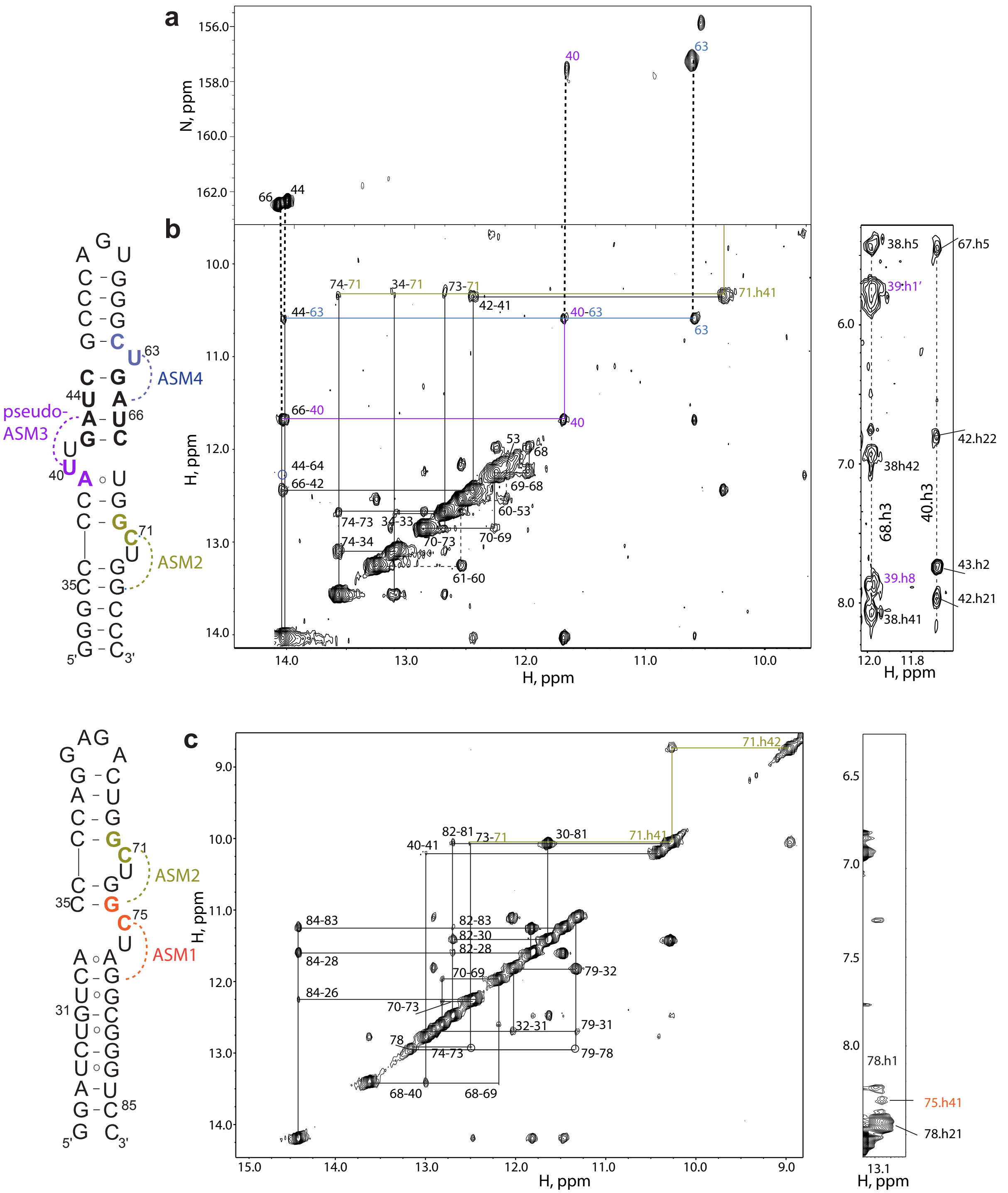

Supplementary Figure 2. Imino assignments 7SK-SL1^{top} and 7SK-SL1^{bottom}.

(a) Portion of the ^{1}H–^{15}N two-dimensional HSQC spectrum for ^{15}N, ^{13}C-labeled 7SK-SL1^{top} showing the imino resonances for uracils and guanosines. The U_{66} and U_{44} imino resonances are found downfield in regions of typical A-U stacked Watson Crick interactions, whereas U_{40} and U_{63} imino resonances have unusually upfield chemical shifts due to their engagement with triple-base formation. **(b)** Portion of the ^{1}H–^{1}H two-dimensional NOESY spectrum showing imino-to-imino NOEs for the free 7SK-SL1^{top} RNA. Imino-to-imino connectivities for ASM_2, pseudo-ASM_3, and ASM_4 are shown in olive, magenta, and

blue respectively. Formation of triple bases in the three ASMs is noted by connectivities between the U_{40} and U_{63} iminos and the C_{71} amino with Watson-Crick base pairs in the stem. Imino connectivities between U_{40} and U_{63} indicate that formation of the pseudo-ASM_3 and ASM_4 triple bases respectively bring the residues close in space. Formation of the reverse Hoogsteen is shown to the right where U_{68} has strong connectivities to A_{39} H8 and H1' protons rather than with the H2 proton seen in regular Watson-Crick interactions. **(c)** Portion of the ^{1}H–^{1}H two- dimensional NOESY spectrum showing imino-to-imino NOEs for the free 7SK-SL1^{bottom} RNA. Imino-to-imino connectivities for ASM_1 and ASM_2 are shown in orange and olive respectively. No change was observed in the formation of the C_{35}-G_{74}:C_{71}^{+} triple base of ASM_2 between the 7SK-SL1^{top} and the 7SK-SL1^{bottom}. Formation of the C_{33}-G_{78}:C_{75}^{+} triple base of ASM_1 is shown to the right with a connectivity between the G_{78} imino and the C_{75} amino. Collectively, the data show that the tandem and the near-symmetrical units fold independently of each other.

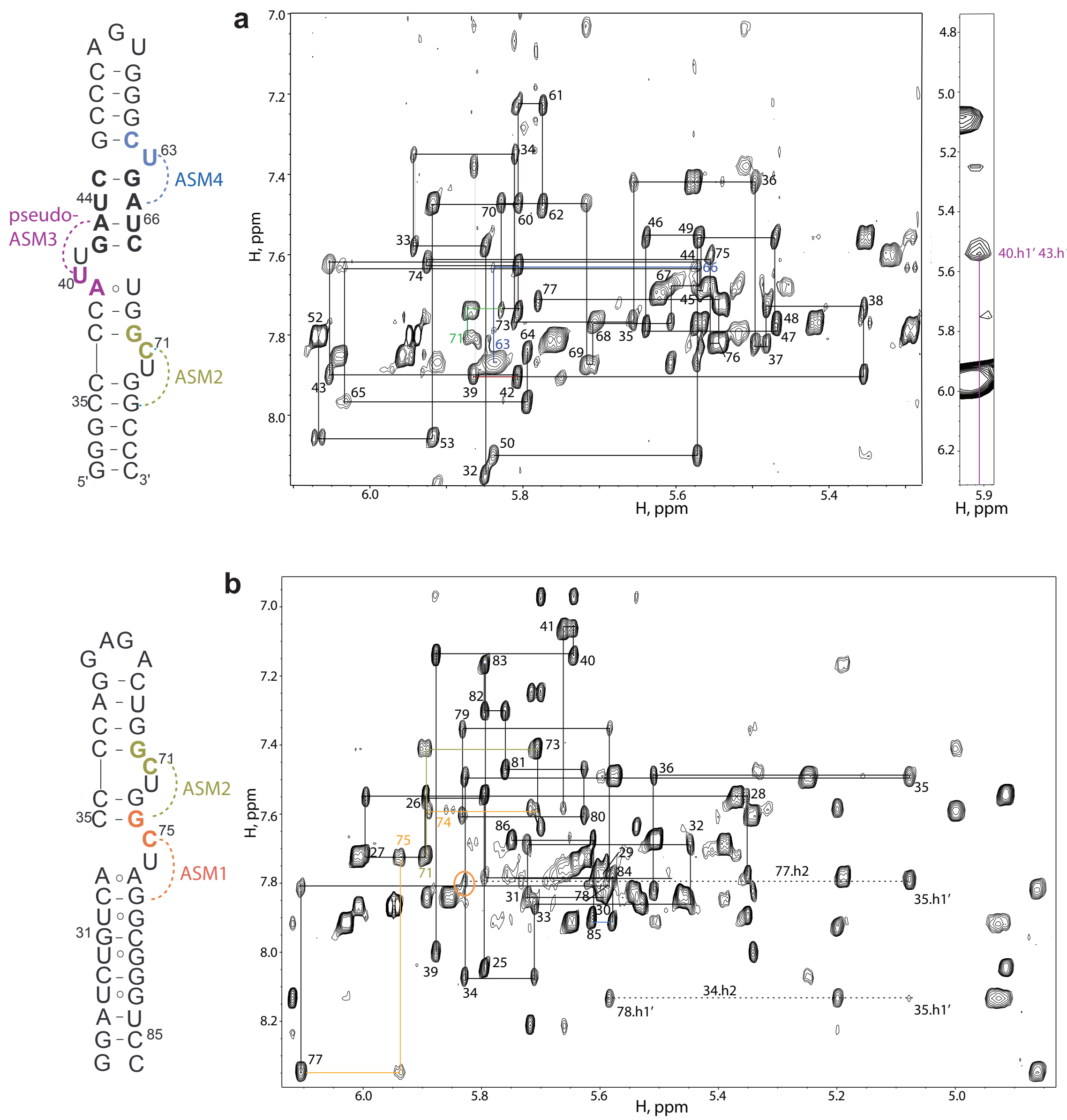

Supplementary Figure 3. Ribose to aromatic connectivities of 7SK-SL1^{top} and 7SK-SL1^{bottom}.

(a) Two-dimensional ^{1}H–^{1}H NOESY spectra for the free 7SK-SL1^{top}. Blue, magenta, and olive lines indicate inter- and intra-residue aromatic-to-H1′ NOE connectivities in ASM_4, ASM_3, and ASM_2 respectively. NOE connectivities between C_{73} and C_{71} (olive) as well as U_{63} and U_{66} (blue) verify the placement of the bulge residue in the same plane as C_{35}-G_{74} and U_{44}-A_{65} respectively. NOE connectivities showing U_{40} triple-base interactions are too overlapped to be observed in this region of the spectra although the examination of the region for H1 -H1 connectivities (right) reveals NOE connectivities between U_{40} H1 and A_{43} H1 protons. **(b)** Two-dimensional ^{1}H–^{1}H NOESY spectra for the free7SK-SL1^{bottom}. Black, orange, and olive lines indicating inter- and intra-residue aromatic-to-H1′ NOE connectivities within the stem. NOE connectivities between C_{75} and A_{77} (orange) as well as G_{73} and C_{71} (olive) verify the placement of the bulge residue in the same plane as C_{33}-G_{78} and C_{35}-G_{74} respectively. The orange circle shows the cross- strand NOE between A_{77} H2 and A_{34} H1' suggesting a hydrogen bonding interaction between the N1 of A_{34} and the amino of A_{77}.

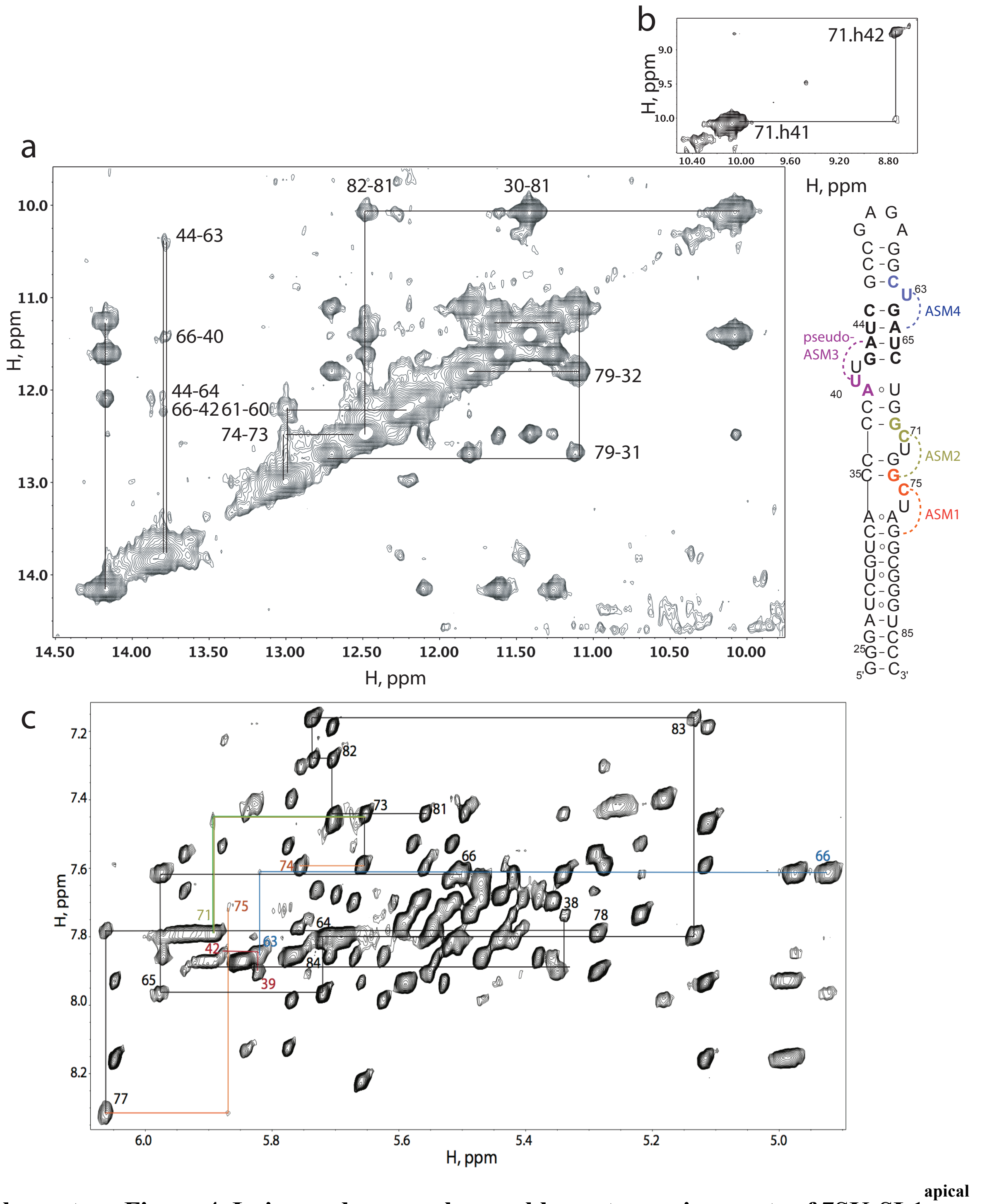

Supplementary Figure 4. Imino and non-exchangeable proton assignments of 7SK-SL1^{apical}.

Panels **(a)** and **(b)** represent the NOESY spectrum showing portions of the ^{1}H–^{1}H two-dimensional imino-to-imino NOEs for the full 7SK-SL1^{apical} RNA. Note that no change in hydrogen-bonding connectivities were observed compared to assignments of the 7SK-SL1^{top} and 7SK-SL1^{bottom} except in the terminal G-C base pairs, demonstrating that the NOE connectivities of the individual upper and bottom constructs is representative of the motifs in the full-length construct. Panel **(a)** shows the long-range NOEs from U$_{66}$ and U$_{44}$ to U$_{40}$ and U$_{63}$, respectively, and panel **(b)** shows the downfield shifted

C_{71}^{+} amino due to its participation in a triple base. **(c)** Two-dimensional ^{1}H–^{1}H NOESY spectra for the free 7SK-SL1^{apical}. Blue, magenta, olive, and orange lines indicate inter- and intra-residue aromatic-to-H1′ NOE connectivities of ASM_4, ASM_3, ASM_2, and ASM_1 respectively. There were no changes in the NOE connectivities that distinguish triple base formation between the different motifs from the assignments of the 7SK-SL1^{top} and 7SK-SL1^{bottom}. Other aromatic-to-H1 NOE connectivities between helical residues within the stem also remained unchanged, demonstrating that the NOE connectivities of the individual upper and bottom constructs is representative of the motifs in the full-length construct.

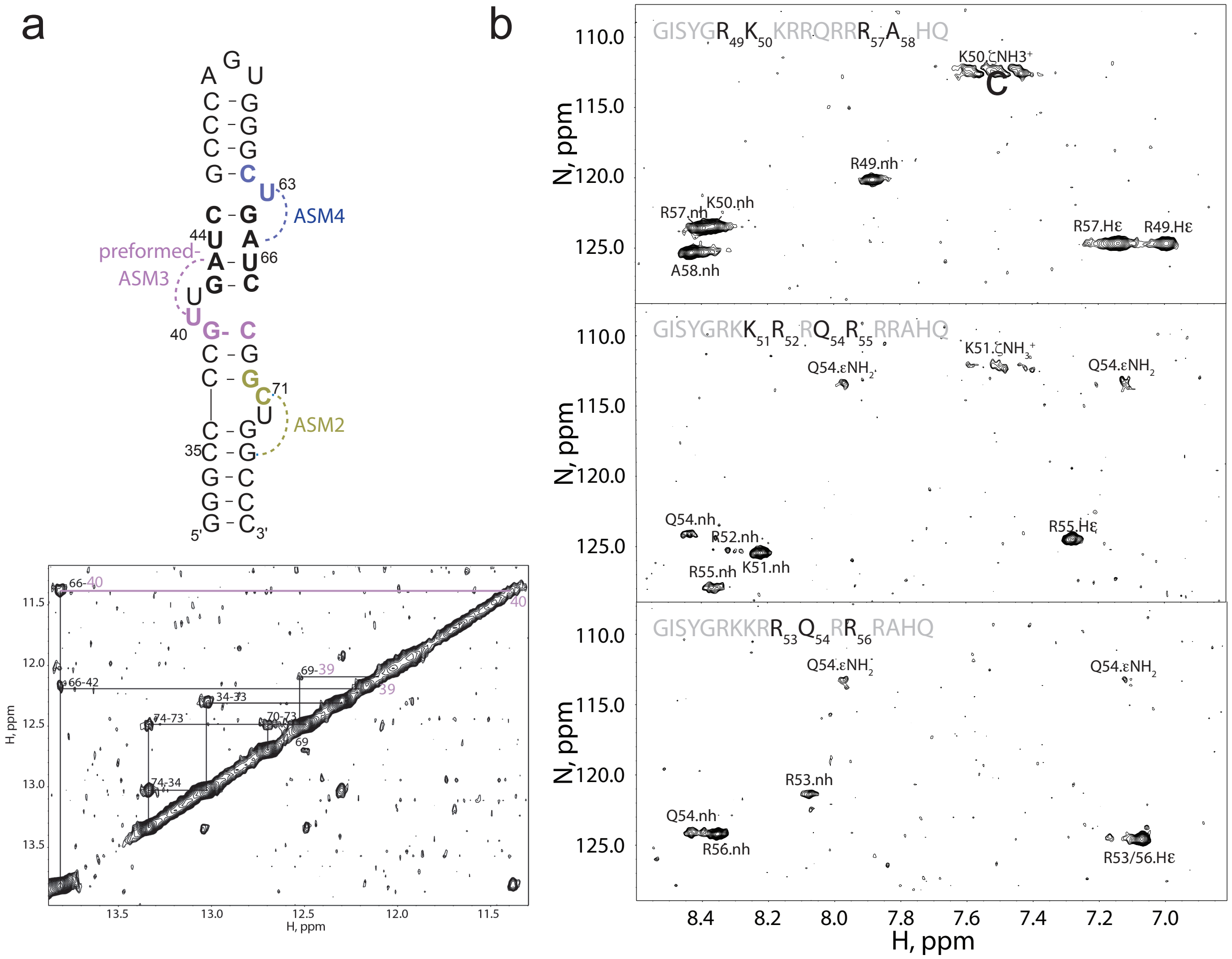

Supplementary Figure 5. Characterization of pseudo-ASM$_3$ and assignments of Tat RBD in complex with 7SK

(a) Portion of ^{1}H–^{1}H two-dimensional NOESY spectra for the A$_{39}$G, U$_{68}$C mutant. As evidenced by the imino-imino connectivity between U$_{66}$ and U$_{40}$, the triple-base cap of ASM$_3$ is still present after replacing the A$_{39}$°U$_{68}$ reverse Hoogsteen with a G$_{39}$-C$_{68}$ (purple) base pair. Also, a regular walk from G39 to G69 shows that G$_{39}$-C$_{68}$ is involved in Watson-Crick interaction. Since there is no change in the chemical shifts of the mutated G$_{39}$-C$_{68}$ upon binding Tat RBD, we conclude that mutating the reverse Hoogsteen to a Watson-Crick base pair converts the pseudo-ASM$_3$ into a classical ASM. **(b)** HSQC spectra of selectively ^{13}C/^{15}N- labeled Tat RBDs upon complex formation. Bolded residues in the sequences are the amino acids that have been selectively labeled. Along with 3D experiments, all backbone amide protons and the side chain amino and guanidinium were unambiguously assigned with the help of this selective labeling strategy. The following shows the HSQC spectra used for protein assignments of Tat RBD in 7SK-SL1^{top}.

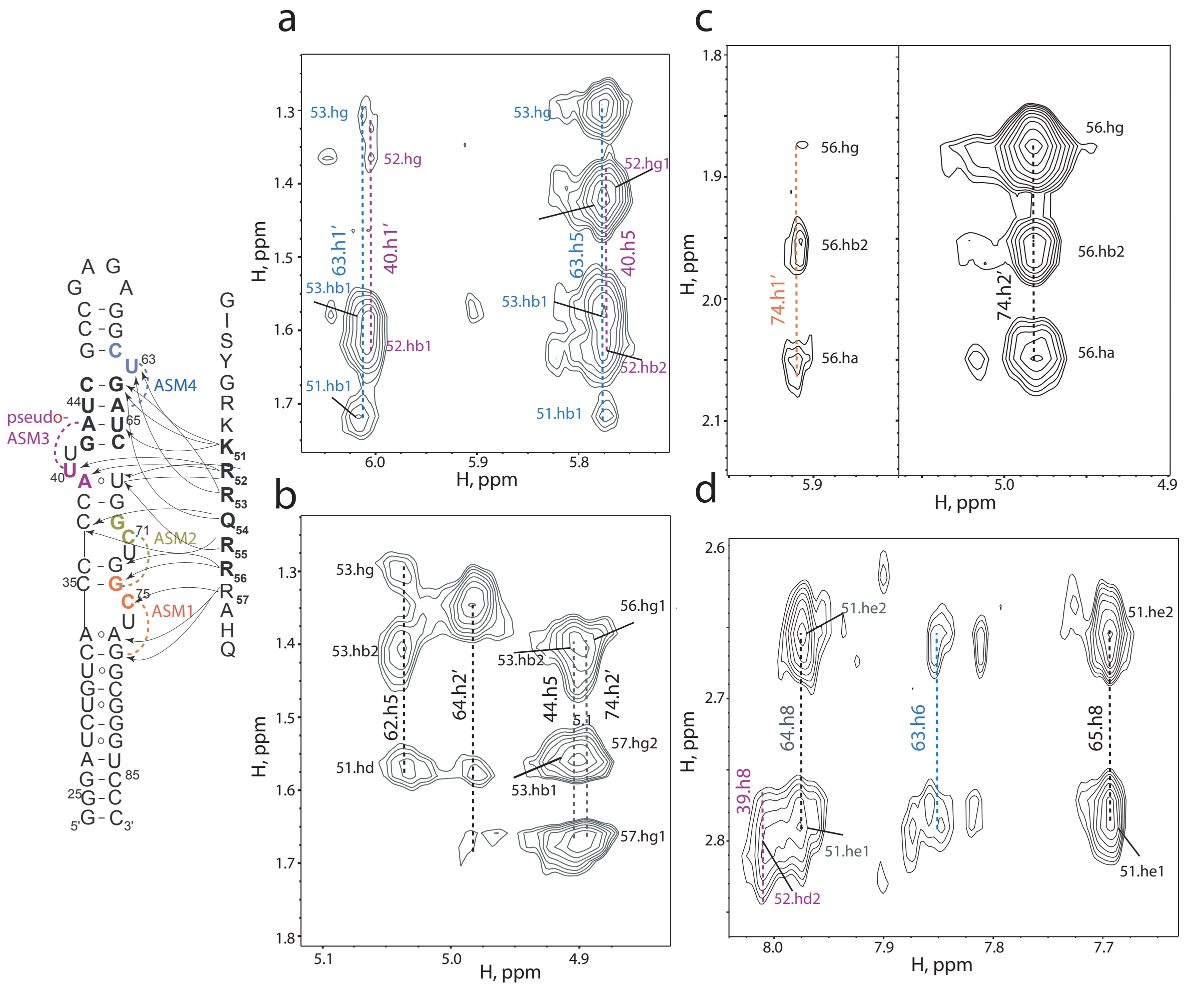

Supplementary Figure 6. Assignments of Tat RBD and 7SK-SL1[apical] complex interaction.

Secondary structure of the 7SK-SL1[apical] with arrows showing NOE connectivities to Tat RBD (for detailed information see also Supplementary Discussion) and portions of ^{1}H–^{1}H two-dimensional NOESY spectra showing representative intermolecular NOEs between Tat RBD and 7SK-SL1. Panel **(a)** shows NOEs between R52 with residue U_{40} and K51 and R53 with U_{63}. Since these two guanosines are involved in base pairs that lie between the cap and the base of the sandwich, these NOEs confirm the intercalation of these arginines into ASM_3 and ASM_4. Panel **(b)** shows NOEs between K51 and R53 with U_{44}, C_{62}, and G_{64} and NOEs between R56 and R57 with G_{74} h2’. Panel **(c)** shows NOEs of R56 with both G_{74} h1’ and h2’protons, placing this arginine into ASM_2 rather than ASM_1. NOEs between R57 and C_{75} and G_{78} confirm the ladder-like configuration of the tandem motifs. Panel **(d)** shows various intermolecular NOEs of K51.

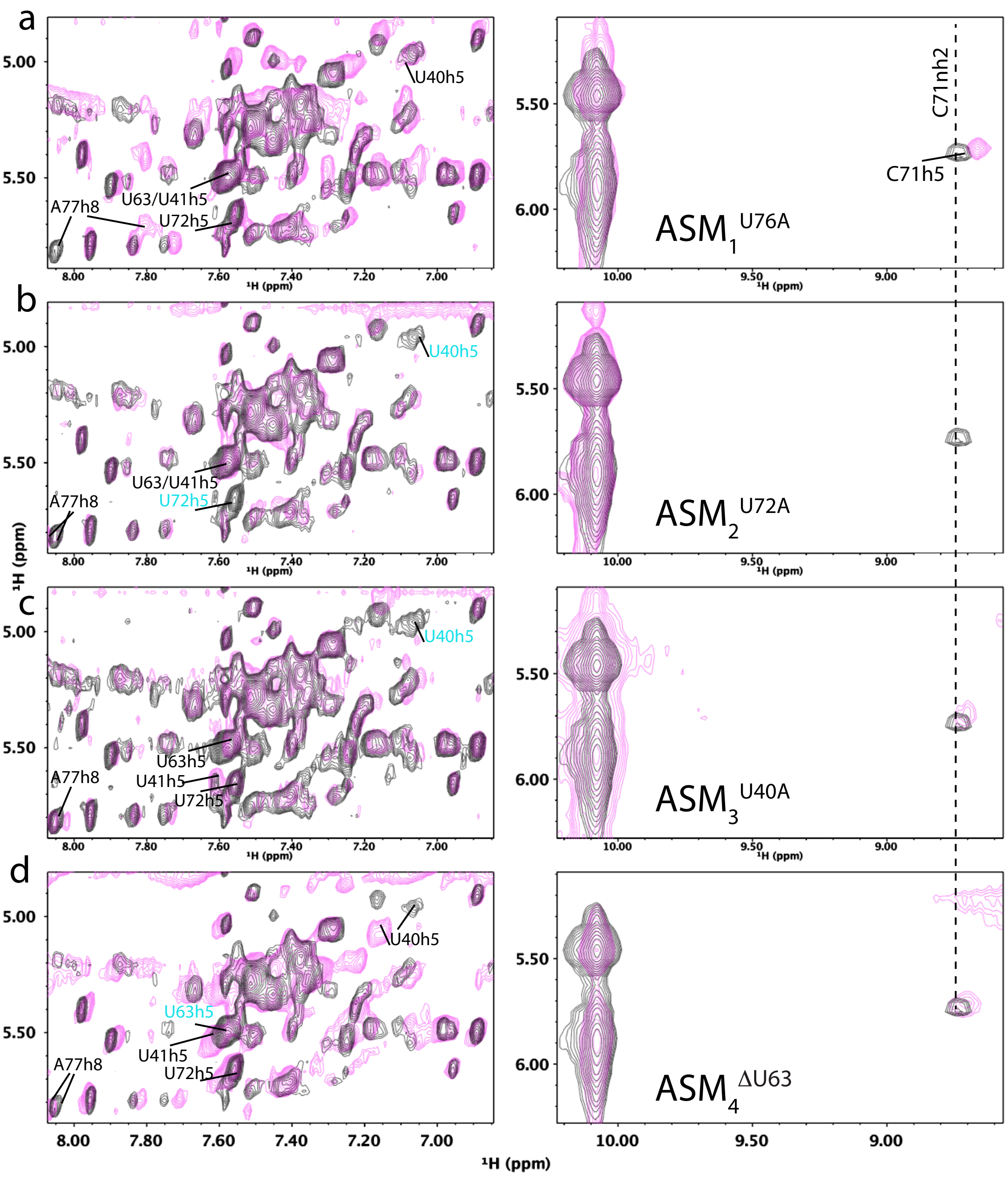

Supplementary Figure 7. Characterization of 7SK-SL1^{apical} mutants.

Portions of the ^{1}H–^{1}H two-dimensional NOESY spectrum for 7SK-SL1^{apical} mutant constructs (magenta) overlaid with that of wild-type (black). Left panels show characteristic shifts of ASM_1, ASM_3, and ASM_4 whereas right panels show the characteristic shift of ASM_2. Abrogation of the mutated ASMs in the respective mutant constructs, and the maintenance of the non-mutated ASMs is evidenced by only local perturbations of: **(a)** the A_{77} H8 in ASM_1, **(b)** U_{72} H5 and the lack of a downfield-shifted $C71^{+}$ amino connectivity with its H5 proton in ASM_2, **(c)** U_{40} H5 proton and movement of the U_{41} H5 proton in pseudo-ASM_3**,** and **(d)** U_{63} H5 proton in ASM_4**.** Lack of proton density due to mutation of residues are indicated by cyan labels.

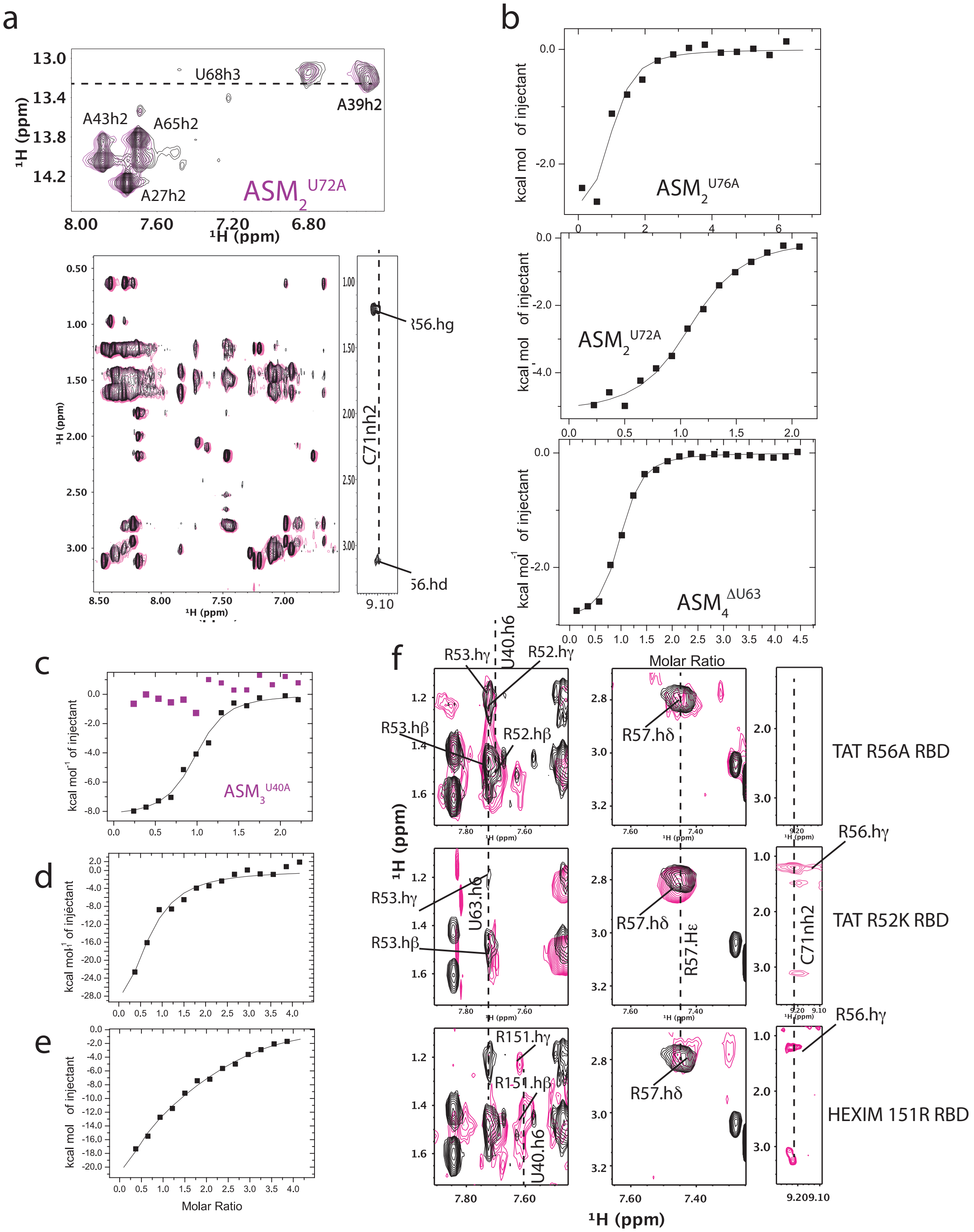

Supplementary Figure 8. Relative contributions of the ASMs and the arginines for the Tat RBD:7SK-SL1^{apical} interaction.

(a) Overlay of two-dimensional ^{1}H–^{1}H NOESY spectra showing that the 7SK-SL1^{apical}-ASM$_2^{U72A}$ mutant (magenta) is capable of switching the pseudo to a classical ASM$_3$ denoted by the appearance of NOE between the U_{68} imino proton to the upfield shifted A_{39} H2 proton in a manner identical to that of

the wildtype construct (black). With the exception of the loss of interactions with ASM_2 (inset), interactions with the other ASMs remain unperturbed. **(b)** Representative ITC data for Tat RBD to an ASM_1 mutant ($U_{76}A$), an ASM_2 mutant ($U_{72}A$), and an ASM_4 mutant (ΔU_{63}). **(c)** Representative ITC data for Tat RBD to an ASM_3 mutant ($U_{40}A$) (magenta). For comparison, the binding curve of Tat RBD to 7SK-SL1^{apical} is overlaid (black). **(d,e)** Representative ITC data for Tat R52K binding to either native 7SK-SL1^{apical} or the ASM_3 preformed $A_{39}G$, $U_{68}C$ mutant, respectively. Tat R52K is able to engage in specific binding with 7SK-SL1^{apical} but not with the $A_{39}G$, $U_{68}C$ mutant, as evidenced by the high N-value. **(f)** Overlay of two-dimensional ^1H–^1H NOESY spectra of wild-type Tat RBD binding to 7SK-SL1^{apical} (black) compared to 7SK-SL1^{apical} binding of mutant constructs (pink). Tat R56A and one conformation of R52K causes the loss of intermolecular NOEs associated with ASM_2 (top) and ASM_3 interactions (middle), respectively. Spectra of titration with HEXIM K151R (bottom) shows engagement of the tandem ASM_1 and ASM_2 motifs by the characteristic shift of the R57 guanidinium moiety, and the intermolecular NOE between the protonated C_{71}^+ amino protons to the R56 gamma protons, respectively. One conformation of the K151R complex shows the engagement of the R151 with ASM_3 as evidenced by NOEs from its beta and gamma protons to the U_{40} sandwich base.

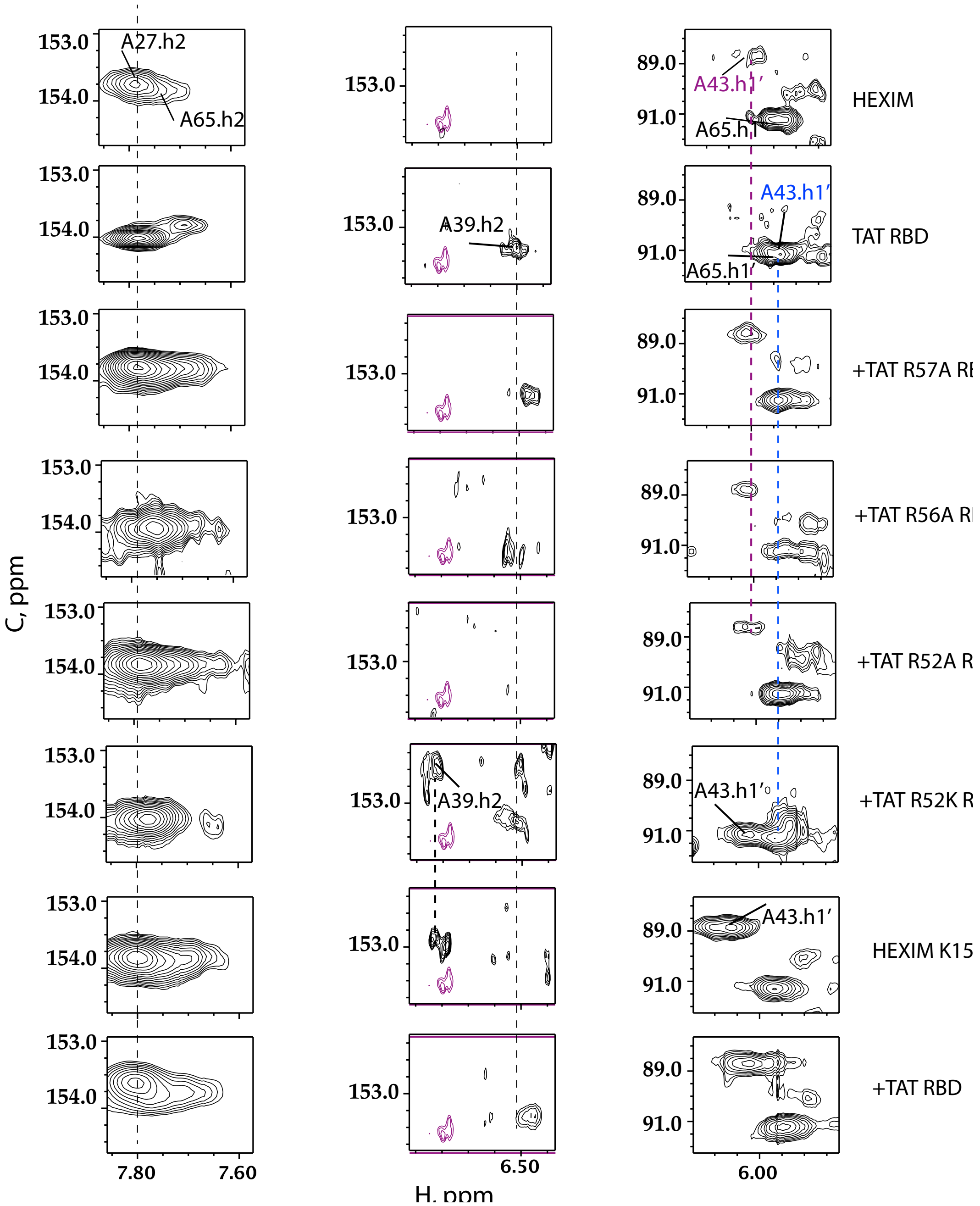

Supplementary Figure 9. Competition experiments of various Tat and HEXIM RBD mutants.

Competition experiments of various Tat RBD mutants (1 equivalents) with HEXIM RBD (2 equivalents) in complex with 7SK-SL1^{apical}. For each titration and competition experiment, the A_{27} and A_{65} H2 proton shifts (left panels) are used as intensity references for comparison with the A_{39} H2 (middle panels) and A_{43} H1' (right panels) chemical shifts, which are used as readouts of HEXIM displacement. The middle panel contains an overlay of the A_{39} chemical shift (magenta) in the free 7SK-SL1^{apical}. R57A and R56A mutants were able to shift the A_{39} H2 proton whereas the R52A mutant was unable to do so. Whereas the shift of A43 H1' indicative of Tat binding (dotted blue line) occurs with titration of the R57A, R56A, and R52A constructs, the continued presence of the A_{43} H1' proton signal characteristic of engagement with HEXIM RBD (dotted magenta line) shows that these constructs do not allow for complete

displacement of HEXIM. On the other hand, competition with an R52K mutant construct was able to efficiently displace HEXIM, although this interaction was dynamic as evidenced by multiple A_{39} H2 and A_{43} H1’ resonances. Finally, titration of 2 equivalents of HEXIM K151R into 7SK-SL1^{apical} was also dynamic, resulting in multiple conformations of the A_{39} H2 proton. Tat RBD was unable to efficiently displace this HEXIM construct as seen by the continued presence of the HEXIM K151R-bound A43 H1’ proton shift.

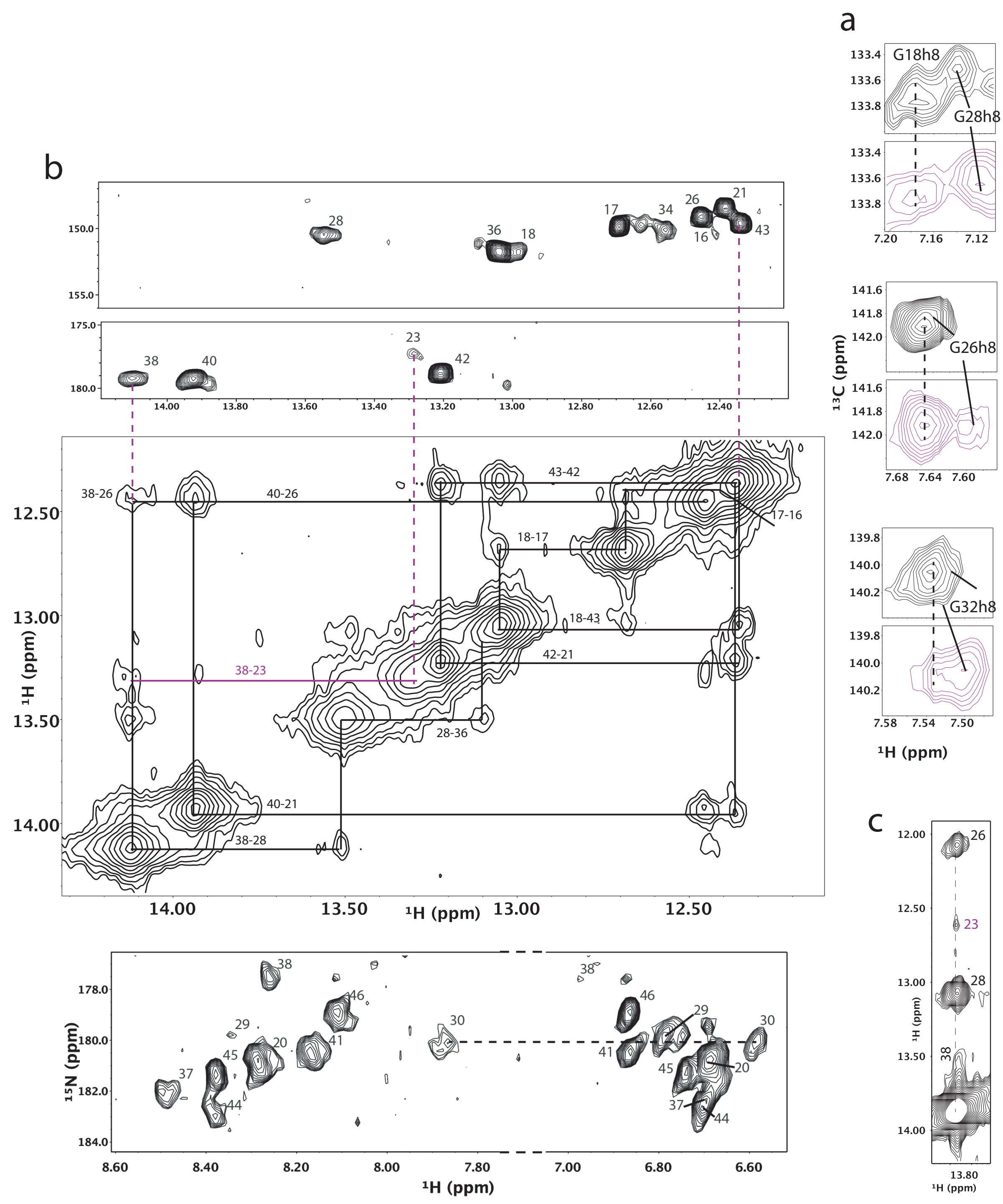

Supplementary Figure 10. Assignments of the Tat RBD:TAR complex.

(a) Titration of ^{13}C-^{15}N G-labeled sample with comparing free TAR (black) to spectra with 0.3 equivalents of CycT1:Tat:AFF4 (magenta) showing the perturbation of the loop residue G_{32} and both the G_{26} and G_{28} residues near the arginine sandwich compared to G_{18}, which does not experience any chemical shift perturbations. (**b**) Portion of the ^{1}H–^{15}N two-dimensional HSQC spectrum for ^{15}N. ^{13}C -labeled TAR RNA showing the imino resonances for uracils and guanosines matched with corresponding portion of the ^{1}H–^{1}H two-dimensional NOESY spectrum showing imino-to-imino NOEs. The U_{38} imino

resonance is found downfield in regions of typical A-U stacked Watson Crick interactions and makes an imino-to-imino connection to the U_{23} imino due to the triple-base formation. Additionally, the emergence of the G_{34} imino indicates the formation of the predicted pseudo-triloop. The presence of this base pair is confirmed by the presence of the C_{30} amino pair in the ^{1}H–^{15}N two-dimensional HSQC spectrum for ^{15}N. ^{13}C -labeled TAR RNA showing the amino resonances of cytosines (dashed line). **(c)** Changing the native loop to a GNRA tetraloop in TAR induces the formation of the pseudo configuration as evidenced by the emergence of the U_{23}-U_{38} imino connection.

21

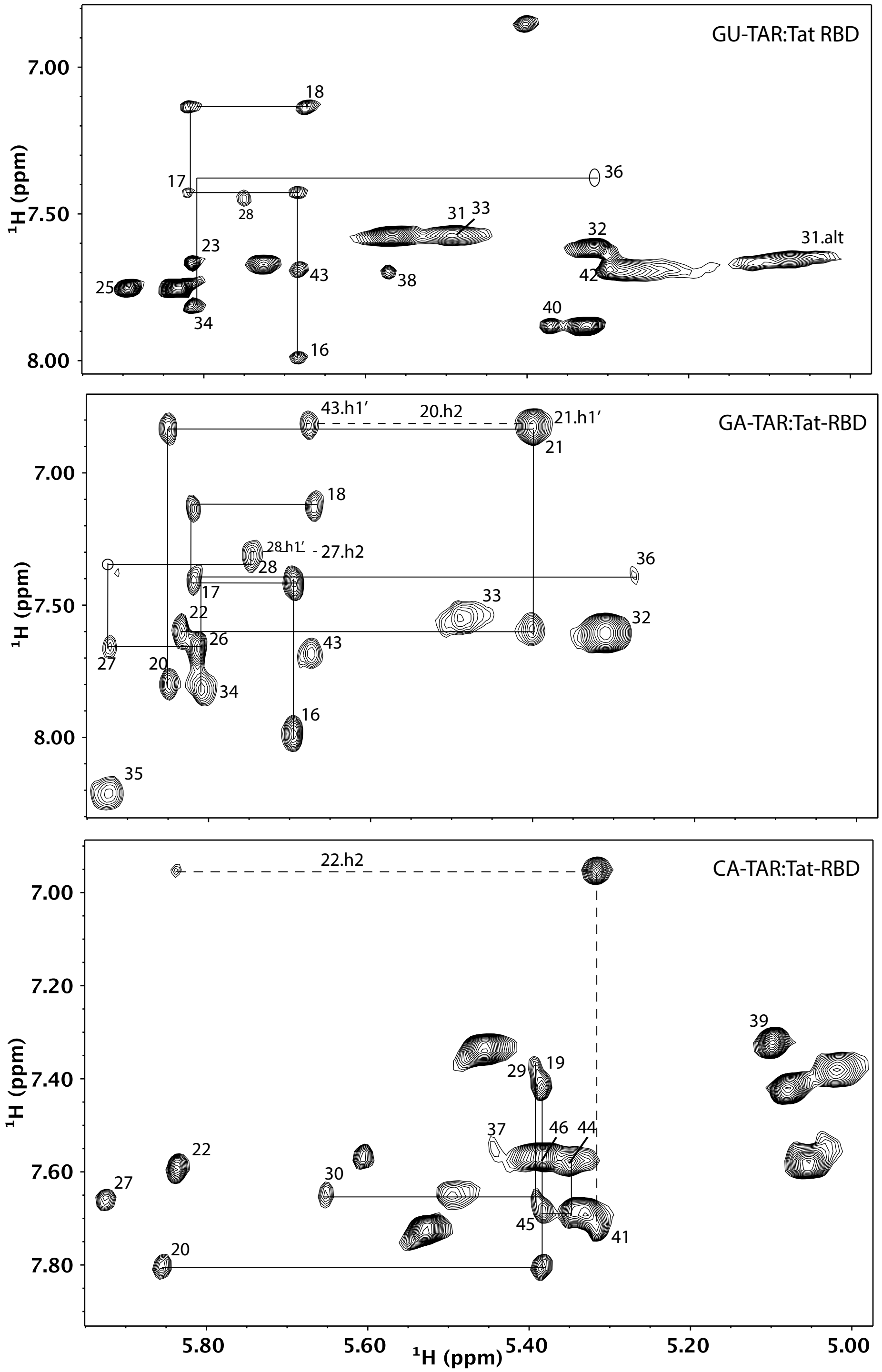

Supplementary Figure 11. Non-exchangeable RNA proton assignments of Tat RBD:TARComplex.

Portion of two-dimensional ^{1}H–^{1}H NOESY spectra of Tat RBD:TAR using GU (top), GA (middle), and AC (lower) protonated samples with the other nucleotides being deuterated.

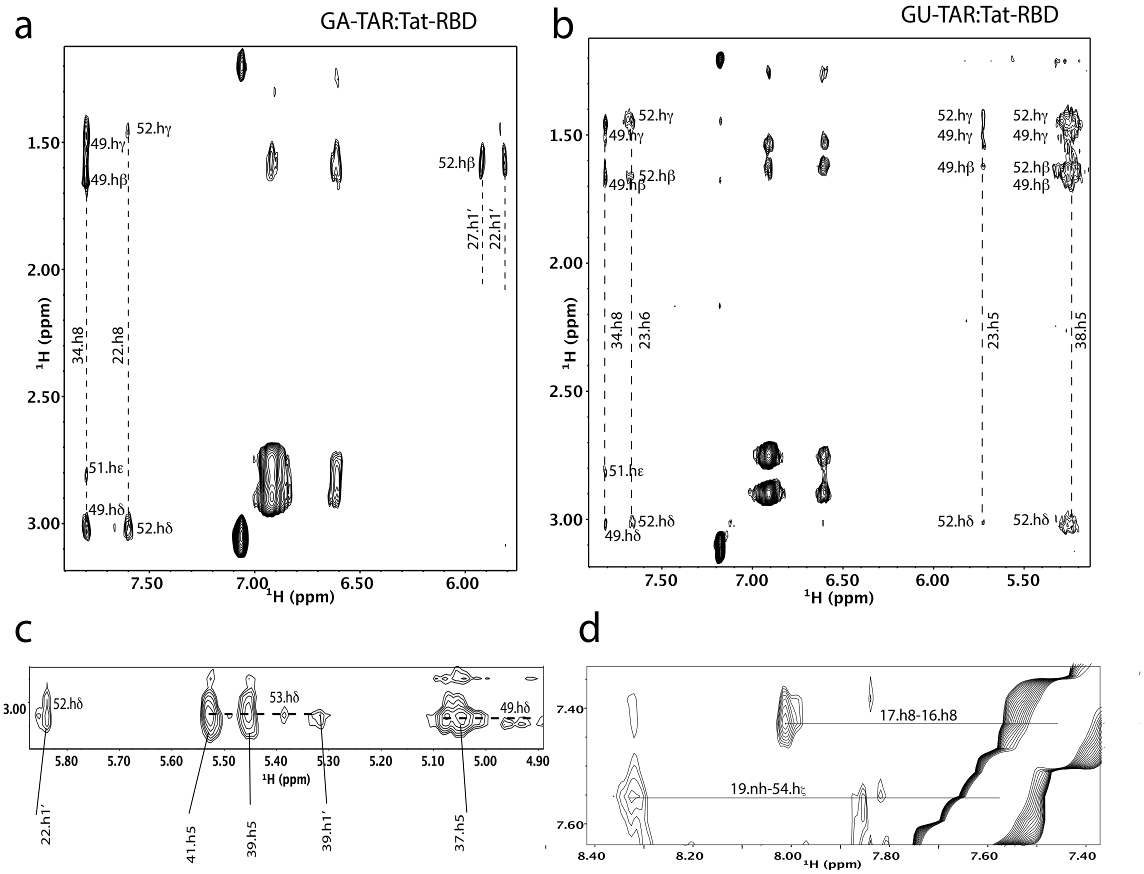

Supplementary Figure 12. Assignment of Tat RBD and TAR complex interaction.

Portions of ^{1}H–^{1}H two-dimensional NOESY spectra showing representative intermolecular NOEs between Tat RBD and TAR. Panel **(a)** shows intermolecular NOEs between Tat RBD and guanosines or adenosines. NOEs are observed between R49 and K51 with G_{34} while R52 interacts with both the A_{22}. Also shown are NOEs from the base triple residue, A_{27} to the R52 β protons. Panel **(b)** shows intermolecular NOEs between Tat RBD and either guanosines or uracils. NOEs can be seen between R49 and G_{34}, U_{23}, and U_{38} and R52 with U_{23} and U_{38}. The NOE connectivities between both the R49 and the R52 protons with both U_{23} and U_{38} place the two arginines above and below the U_{23} sandwich cap. Panel **(c)** shows intermolecular NOEs between the R53δ proton and C_{39} and C_{41}. **(d)** Portion of two-dimensional ^{1}H–^{1}H NOESY spectra showing NOEs between the Q54 h ζ protons and the C_{19} amino protons. As there are no NOEs between R55 and TAR, this positions Q54 as the C-terminal exit from TAR.

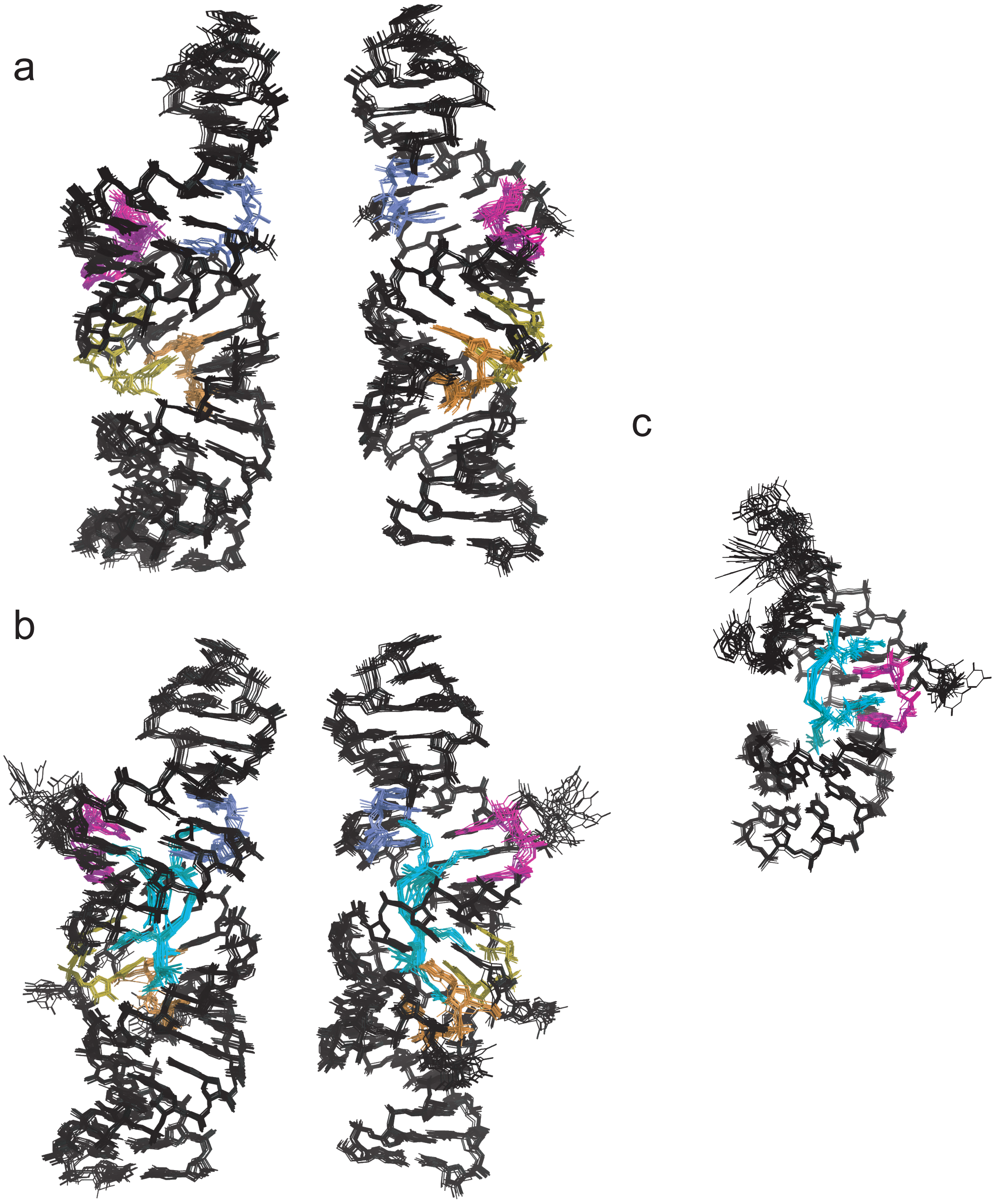

Supplementary Figure 13. Ensembles of the low-energy structures

(a) Two views of the twelve low-energy conformers of free 7SK-SL1^{apical} without U_{41}, U_{72}, and U_{76} bulge residues, which are dynamic and flexible. In keeping with the main text, ASM_1-ASM_4 are colored, orange, olive, magenta, and blue, respectively. (**b**) Two views of the twelve low-energy conformers of 7SK-SL1^{apical} bound to Tat RBD (cyan). Flexible N-terminal residues 44-49 and C-terminal residues 59 and 60 are not shown. (**c**) Ensemble of the twelve low-energy conformers of TAR bound to Tat RBD (cyan, right). The arginine sandwich motif is shown in magenta. Flexible N-terminal residues 44-47 and C-terminal residues 56-60 are excluded from the ensemble.

Supplementary Discussion

Free 7SK-SL1^{apical}

It is important to comment on the difference between our solution structure of the free 7SK-SL1^{apical} to the one that was recently solved by Bourbigot et al[1]. Like Bourbigot et al, we see that at low osmolarity, ASM_2, pseudo-ASM_3, and ASM_4 do not form and the residues that would have formed the sandwich caps (C_{71}, U_{40}, and U_{63}, respectively), instead, are involved in stacking interactions with the preceding bulge residues. However, under these conditions, Tat and HEXIM RBDs bind non-specifically to 7SK-SL1^{apical}; leading us to conclude that the structure solved by Bourbigot et al. may not be the physiological state of the molecule[1]. Our structure, on the other hand solved at higher osmolarity, shows how the four ASMs are configured to properly engage HEXIM and Tat RBDs as evidenced by highly specific 1:1 binding. Similarly, our free structure differs with the one solved by Martinez-Zapien et al. at the asymmetric motifs where the major regulatory activity occurs[2]. In their structure, the U_{63} makes a triple base pair with the G_{42}-C_{67} base pair: we do not observe such a conformational state neither in the free state nor upon Tat or HEXIM RBD binding.

The imino spectra of 7SK-SL1^{top} matched the expected secondary structure; we observe the three distinctive alternating G^{o}U base pairs in the lower part of the stem-loop along with the expected Watson-Crick interactions. Under higher salt concentrations (70 mM NaCl) we observed a network of additional imino connectivities in regions that are typical of non Watson-Crick hydrogen bonding interactions. Assignments of these connectivities indicated long-range interactions from the bulges C_{75}U, C_{71}U, U_{40}U and U_{63} to the stem regions. Several unambiguous base-ribose NOE contacts place residues that form the roofs of the sandwich in an approximately planar orientation to the n+1 Watson-Crick base pair with a potential to form a base triple interaction (Supplementary Figures 2-4).

1) ASM_2: The C_{71}^{+} base is protonated as evidenced by the typical downfield shift of the N4 amino protons (Supplementary Figure 2b), which give NOE interactions with the imino protons of G_{34}, G_{73} and G_{74}, the guanosine residues involved with the three base pairs following the C_{71}^{+} bulge residue. The C_{71}^{+} ribose H1′ has NOE interactions with both the H1 imino and H8 aromatic protons of G_{73}, placing C_{71}^{+} in a planar orientation with the C_{35}-G_{74} base pair. This is additionally confirmed by complementary NOE interactions between the C_{71}^{+} and C_{35} N4 amino protons. Furthermore, an NOE between the C_{71}^{+} amino proton and the H2′ proton of residue C_{36} places the C_{71}^{+} bulge residue into the major groove. Interactions between both the ribose H2′ and H3′ of U_{72} with the G_{73} aromatic H8 places U_{72} in the minor groove facing the proceeding C_{37}-G_{70} base pair. Unlike the surrounding stem residues, both the bulge residues C_{71}^{+} and U_{72} have a C2′ *endo* pucker.

2) ASM_1: The C_{75} residue of the C_{75}U bulge also has a similar orientation as the above C_{71}^{+} base; however, in this case the C_{75} base is not protonated. The presence of the C_{75} amino proton pair with typical chemical shifts (one upfield and another downfield by about 1.5 ppm) indicates that this moiety is involved in hydrogen bonding. A long-range NOE from the downfield amino proton to the G_{78} imino H1 proton puts the residue in a flipped-out orientation in the major groove and in close proximity to the C_{33}-G_{78} base pair (Supplementary Figure 2b). The secondary structure of ASM_1 is different from the others in that the triple base is not preceded by a canonical Watson crick base pair but with a A_{34}, A_{77} bulge. These residues, however, are stacked inside the helix, which allows the sandwich to form without any major differences in the three-dimensional configuration. Specifically, the A_{77} has a typical base stacking orientation as evidenced by intense H2 NOEs to the C_{35} and G_{78} H1′. Similarly, the stacking of

A_{34} between C_{33} and C_{35} is confirmed by a typical NOE walk in this stretch (Supplementary Figure 3b). However, the A_{34} does not give the expected H2 connectivities in that it has weak NOEs to the C_{35} and G_{74} H1′ protons, indicating that A_{34} slides towards the minor groove, placing the N3 acceptor of A_{34} in close proximity to the N6 amino proton of the A_{77}. In support of this potential base-pairing pattern, we see a significant cross-strand NOE between the H2 proton of A_{77} and the H1' proton of A_{34} (Supplementary Figure 3b). The planar orientation of the C_{75} with the C_{33}-G_{78} base pair is confirmed by NOE connectivity from A_{77} H8 to C_{75} H1′.

3) ASM_3: The structure of ASM_3 adopts a pseudo-arginine sandwich motif in the unbound state due to the reverse Hoogsteen interaction in the base of the sandwich. Formation of the reverse Hoogsteen is evidenced by strong connectivities of the U_{68} imino proton to A_{39} H8 and H1' protons rather than with the H2 proton seen in regular Watson-Crick interactions. Although we observe the typical ribose to base NOEs between A_{39} and C_{38} indicating stacking of A_{39}, the absence of NOEs from A_{39} H2 to G_{42} indicates that this residue is shifted towards the minor groove providing additional evidence for the Hoogsteen interaction (Fig. 2c). However, the cap of pseudo-ASM_3 still forms a triple base interaction as evidenced by NOE connectivities between the U_{40} and A_{43} H1′ protons and the interaction between the imino proton of U_{40} with the H2 proton of A_{43}. Complementary NOE interactions between the U_{40} and U_{66} imino protons place U_{40} in a planar orientation with the A_{43}-U_{66} base pair (Supplementary Figure 2b) as do connectivities between the H1' of A_{43} and U_{40}. Additionally, the close special orientation of U_{40} and U_{63} is evidence by connectivities between their iminos (Supplementary Figure 2a, b).

4) ASM_4: The structure of ASM_4 forms a preformed cavity very similar to ASM_1 and ASM_2 despite the presence of a single residue bulge. The U_{63} ribose H1′ show NOE interactions with the H8 aromatic protons of U_{66} while its H5 show NOE interactions with the H1′ of A_{65} (Supplementary Fig 3b). Additionally, a direct imino to imino connectivity between U_{44} and U_{66} confirms the U_{63}:U_{44}-A_{65} triple base interaction.

7SK-SL1^{apical}:Tat RBD Complex

Upon titration of Tat RBD into 7SK-SL1^{apical}, the imino spectra revealed that the overall RNA architecture is maintained. The G^{o}U base pairs in the lower part of the stem-loop as well as the pentaloop residues are not perturbed and do not give any intermolecular NOEs. The protonation of the ASM_2 cap C_{71}^{+} is also maintained as evidenced by the downfield shifted amino pairs (Fig. 2d). Similarly, residues involved in the formation of ASM_1, ASM_2, and ASM_4 showed similar intraresidue connectivities to the free 7SK-SL1^{dsital}, indicating that the preformed nature of these motifs is preserved. For example, the characteristic C_{71}^{+} cap H1′ in ASM_2 maintains the NOE interactions with both the H1 imino and H8 aromatic protons of G_{73}, which continues to place C_{71}^{+} in a planar orientation with the C_{35}-G_{74} base pair. The major changes are observed in ASM_3. For example, the H2 proton of A_{39} experiences a large upfield chemical shift change (0.6 ppm). Its base-pairing partner U_{68} now gives the characteristic imino to A_{39} H2 connectivity, indicating a transition to a Watson-Crick interaction (Fig. 2c). Additionally, binding of Tat RBD also caused the A_{43} H2 to experience a downfield chemical shift compared to A_{65} (in the free 7SK-SL1^{apical}, these two H2 protons had very similar chemical shifts due to the palindromic nature of the triple bases). This indicates that the binding of Tat RBD leads to the global remodeling of pseudo-ASM_3 into a true sandwich motif. Residues within the four ASMs showed intermolecular connectivities to the Tat RBD detailed below.

To unambiguously assign the arginines, we synthesized three peptides with different combinations of $^{13}C/^{15}N$ labeled amino acids (Supplementary Figure 5b). Remarkably, we observed guanidinium nitrogen moieties from all labeled arginines after complex formation, indicating the slow-exchange of these Hη protons due to their involvement in hydrogen-bonding interactions within the sandwiches. The arginine side chains are precisely anchored by interactions between the arginine side chain protons with the aromatic H5/H6 protons of the sandwich roofs. For example, R53 and R52 Hβ and Hγ protons gave strong NOE connectivities to the U_{63} and U_{40} H5 protons, respectively (Supplementary Figure 6a). The overlap in the U_{63} and U_{40} proton chemical shifts (H5, H6, and H1′) indicate that these residues are positioned in a similar chemical environment due to the symmetrical nature of ASM_3 and ASM_4. Similarly, intense NOEs from R52 and R53 Hβ protons to G_{42} and G_{64}, which are involved in Watson-Crick pairs positioned in-between the cap and the base of ASM_3 and ASM_4 respectively, show that these arginine side chains are coplanar with these residues (Supplementary Figure 6a). This positions the arginines in a stacked conformation between the cap and the base of the sandwiches. Similar intermolecular NOEs between R57 and R56 protons to residues within ASM_1 and ASM_2 respectively are observed. Interestingly, the side chain protons of R56 experience uncharacteristic proton chemical shifts due to their stacking below the protonated C_{71}^{+} cap (Supplementary Figure 6c).

The placement of the arginines within the sandwiches is further stabilized by hydrogen bonds from its guanidinium moieties. For example, the planar orientation of the G_{42}-C_{67} base pair with the two amide groups of R52 allows the guanidinium protons to be involved in an arginine fork interaction: one is within hydrogen bonding distance of the O6 of G_{42} while the other interacts with the backbone phosphate O5′ of U_{40} (Fig. 3a). Together, these interactions create a stabilized hydrogen-bonding interaction for R52, which stacks on the U_{40}:A_{43}-U_{66} triple platform and forms an intimate interface optimized by intermolecular electrostatic interactions.

SUPPLEMENTARY REFEREENCES

1. Bourbigot, S. et al. Solution structure of the 5'-terminal hairpin of the 7SK small nuclear RNA. *RNA* **22**, 1844-1858 (2016).
2. Martinez-Zapien, D. et al. The crystal structure of the 5 functional domain of the transcription riboregulator 7SK. *Nucleic Acids Res* **45**, 3568-3579 (2017).
